# Acceptance of Sexual Interest in Minors in Self-Referred Individuals Under Treatment – An Exploratory Pilot Study

**DOI:** 10.3389/fpsyg.2021.606797

**Published:** 2021-11-04

**Authors:** Ute Lampalzer, Safiye Tozdan, Fritjof von Franqué, Peer Briken

**Affiliations:** Institute for Sex Research, Sexual Medicine and Forensic Psychiatry, University Medical Center Hamburg-Eppendorf, Hamburg, Germany

**Keywords:** child abuse material, pedophilia, prevention of child sexual abuse, psychotherapy, risk of offending

## Abstract

Some therapists/scientists argue that “acceptance” of sexual interest in minors (SIM), i.e., the integration of the sexual preference into the individual self-concept, is a prerequisite for dealing with SIM in a responsible way. However, if one assumes that – even in some persons – SIM might change over time, “acceptance” could also run counter to therapeutic targets because the motivation to change as well as the specific self-efficacy for modifying SIM might be reduced. This exploratory pilot study analyzes the relationship between acceptance of SIM and (1) dynamic risk for contact sexual reoffending, (2) SIM and frequency of the use of child/adolescent (sexual abuse) imagery, (3) frequency of sexual desire/behavior toward children/adolescents, and (4) the change of the level of acceptance of SIM during the course of treatment. The majority of the participants (*N* = 79) was not exclusively interested in children (85%) and used child pornography but did not commit child sexual abuse (54%). Acceptance of SIM, frequency of the use of child/adolescent (sexual abuse) imagery and frequency of sexual desire/behavior toward children/adolescents are assessed via self-report questionnaires, dynamic risk for contact sexual reoffending is measured by STABLE-2007. Pretreatment data are analyzed via Spearman’s correlation (*N* = 79). Intragroup analysis compares acceptance of SIM from pre- and posttreatment (*n* = 35). There was no correlation between acceptance of SIM and dynamic risk for contact sexual reoffending. However, there was a medium, positive correlation between acceptance of SIM and the frequency of the use of legal imagery of children, a positive correlation between the item “My inclination is an integral part of my personality” and the frequency of the use of legal imagery of children, and a positive correlation between acceptance of SIM and the frequency of sexual activities with minors. Acceptance of SIM did not change during the course of treatment. The results suggest that “acceptance” of SIM has to be discussed in a differentiated way, i.e., as possibly being associated with positive and negative outcomes as well.

## Introduction

Findings from an online survey with 8,718 German males indicate that 4.1% have sexual fantasies about children and that 3.2% have offended against prepubescent children. But only 0.1% reported a pedophilic sexual preference (Dombert et al., 2016). The sites of the German network “Kein Täter werden” (means: not become an offender) offer treatment for people seeking therapeutic help because of sexual interest in minors (SIM) and distress or a risk of sexual (re-)offending (Netzwerk “Kein Täter werden”, 2018). It is a formal requirement in this network that individuals who receive treatment are currently not in contact with the criminal justice system.

The construct “acceptance,” as it is understood in this exploratory pilot study, means that sexual preference is accepted as a “stable and therefore constantly challenging part of the own personality” which, being “fate rather than choice,” “cannot be changed by any treatment” (Institute for Sexology and Sexual Medicine of the Charité, 2013, pp. 65–66). Going further, it means “the recognition of reality […], one’s way of easing the pain, when realizing that things are unchangeable […] [, and] abandonment of the wish to change given reality” (Institute for Sexology and Sexual Medicine of the Charité, 2013, p. 67). Thus, it goes along with an ego syntonic concept of sexual preference which allows therapy right away to focus on aspects other than sexual preference (Institute for Sexology and Sexual Medicine of the Charité, 2013).

Some therapists and scientists argue that “acceptance” of pedophilic interest is needed for individuals with sexual interest in minors (SIM) for dealing in a responsible way with their sexual interest and thus preventing sexual abuse (Ahlers et al., 2008; Institute for Sexology and Sexual Medicine of the Charité, 2013). Other therapists and scientists, however, argue that – even in some persons, and maybe more often in men with non-exclusive pedophilia (Tozdan and Briken, 2019) – SIM might change over time and that “acceptance” might run counter to prevention goals because motivation to change as well the specific self-efficacy for modifying sexual interest in children may be reduced by it (Tozdan and Briken, 2015a; Fedoroff, 2020).

Seto (2012) argues that sexual orientation is characterized by an age of onset before the beginning of puberty and by stability over time, and that pedophilia is similar in these respects. He refers to findings on identified and self-identified child sexual abuse offenders with pedophilia of whom a considerable proportion report an age of onset of sexual interest in children before adulthood (e.g., Li, 1991; Marshall et al., 1991; Freund and Kuban, 1993). Furthermore, he cites studies which suggest that pedophilia is predictive for future sexual behavior involving children even one to three decades later (Hanson et al., 1993), and that treatment-related decrease of sexual arousal related to children does not reduce recidivism rates (Rice et al., 1991), i.e., does not persist (Seto, 2012). Due to these findings, he makes the case for “conceptualizing pedophilia as a type of sexual orientation in males” (Seto, 2012, p. 231). Cantor (2018) also takes the position that pedophilia is immutable. He claims that lines of indirect evidence referring to the following “all have much more parsimonious and mundane explanations” (Cantor and Fedoroff, 2018, p. 205): “Sex crime rates are dropping […]. The incidence of sex crime rates decreases as people age […]. The likelihood that (known) high-risk sex offenders will re-offend decreases the longer they commit no crime […]. The self-report of men and women with paraphilic disorders […]. As people grow older, their interests shift to partners similar in age” (Cantor, 2018, p. 205). He argues that these findings reflect “aging populations,” “the decrease in sex drive that accompanies aging,” “the absence of critical thinking,” and “sexual behavior with partners of increasing age as they [people] themselves age” (Cantor, 2018, p. 205). Researchers who assume that pedophilia is immutable usually infer that treatment should “focus on […] management” of pedophilia (Seto, 2017, p. 18), i.e., on developing the skills that are needed to regulate and control pedophilic urges (Institute for Sexology and Sexual Medicine of the Charité, 2013; Lehmiller, 2019).

Marshall (2008) assumes that pedophilia is not immutable because after treatment phallometric data show a reduction of arousal to children and an increase of arousal to adults in “quite deviant child molesters” (Marshall, 2008, p. 42). Marshall et al. (2009) describe behavioral procedures for modifying sexual interests, i.e., different aversion and masturbatory-based techniques, and the evidence base for these procedures. Marshall et al. (2011) opt for identifying “individualized appropriate (i.e., non-deviant) sexual scripts that can serve as both templates for actual sexual relations and as images for masturbation activities” and refer to the fact that “[U]unreinforced habitual behavior (in this case, deviant sexual interests) has been shown to extinguish such habits” (Marshall et al., 2011, p. 152). Recent research on age of onset of sexual interest in children with individuals from different contexts (with and without treatment, explicitly and not explicitly advocating against acting on sexual interest in children) shows broad ranges from 6 to 44 years and 7 to 66 years, with a mean value of 17 and 20 years (Tozdan and Briken, 2015b, 2019). It also shows that a later age of onset is associated with more perceived flexibility of sexual interest in children, that more perceived flexibility is related to more motivation to change sexual interest in children (Tozdan and Briken, 2019), and that an increasing specific self-efficacy for modifying a sexual interest in children is related to a decreasing sexual interest in children in a considerable number of individuals (Tozdan et al., 2018b)^1^. Researchers who assume that pedophilia is mutable usually conclude that therapeutic interventions should target patients’ specific self-efficacy (Briken et al., 2014; Tozdan and Briken, 2015a; Tozdan et al., 2018a), empower them (Fedoroff, 2018), and work on “relationship skills and healthy sexuality […], self-esteem, empathy, prosocial sexual attitudes, and coping skills” (Marshall et al., 2011, p. 152). Briken et al. (2018) argue that, under the assumption of mutability of deviant sexual interests in at least a part of patients, therapists should clarify what their patients’ motivation and goal in treatment is. As many patients do not have a desire for change, therapists should be open to adapt treatment to assignment, risk of (re-)offending, and exclusiveness of SIM (Briken et al., 2018).

In summary, at the present stage of research, one can assume that there are patients in which SIM might change over time or not, depending on, for example, flexibility, exclusivity, and age of onset of SIM (Tozdan and Briken, 2019). Hence, it can be expected that acceptance is a differentiated construct, too.

## Study Aim

We can safely assume that a therapist’s attitude concerning the necessity of accepting SIM is considered rather relevant for the therapeutic process. Indeed, it already has been shown that there is a relation between therapists’ attitude toward the immutability of SIM and their patients’ self-efficacy to change their SIM (Tozdan et al., 2018a). That means, the more therapists are convinced that SIM is mutable, the more their patients believe they can change it and vice versa. It is lively debated if patients should be told that SIM can change, if patients should be told that SIM is unchangeable (Cantor, 2018; Cantor and Fedoroff, 2018; Fedoroff, 2018), or if this question can only be answered in the course of the therapeutic process, because there are very different courses of SIM.

What we do not know is if the acceptance of SIM is related to patients’ motivation to control sexual urges, to change their SIM, or to behavioral treatment outcomes, such as consumption of sexual imagery of minors and/or sexual abuse of minors. Furthermore, it has not yet been studied if there is a difference between acceptance of SIM and behavior outcomes that correspond to pedophilic or to hebephilic interests. We also do not know if acceptance of SIM is related to dynamic risk factors which are linked with sexual self-regulation (Hanson et al., 2007). Moreover, it has not yet been clarified if acceptance of SIM is associated with sexual desire. Furthermore, we do not know if acceptance of SIM changes in the course of treatment, i.e., is affected by treatment.

Therefore, the purpose of this exploratory pilot study is to investigate the following research questions:

(1) Is there a relationship between the acceptance of SIM and dynamic risk factors?
(2) (a) Is there a relationship between the acceptance of SIM and the frequency/intensity of the use of child abuse/exploitation material? (b) Is there a relationship between the acceptance of SIM and the frequency/intensity of the use of adolescent abuse/exploitation material?
(3) Is there a relationship between the acceptance of SIM and the frequency/intensity of sexual desire/behavior toward minors?
(4) Does the level of acceptance of SIM change in the course of treatment?

## Materials and Methods

### Participants

This exploratory pilot study included 84 adult men with SIM who underwent initial diagnostic procedures between autumn 2011 and autumn 2019, gave their informed consent, and started treatment at the Institute for Sex Research, Sexual Medicine and Forensic Psychiatry in Hamburg. Thirteen men who had not given their informed consent were not included. The study was approved by the Ethics Committee of the Chamber of Psychotherapists Hamburg (09/2019-PTK-HH, 02/2015-PTK-HH). Data were prepared for analysis by two researchers (UL and ST) working at the research unit of the institute. Their work was independent of the processes of treatment indication and psychotherapeutic care.

All of the participants fulfilled the preconditions for receiving treatment in the program offered by the Prevention Network “Kein Täter werden” (see Table 1 one for demographic characteristics of the participants). These are:

**TABLE 1:** Sample characteristics for the total sample (*N* = 79) when undergoing initial diagnostic procedure.

Variables	Total (*N* = 79, 100%)	
	*N* ^a^	%^b^
**Education level**		
Less than 10 years	13	16.5
More than 10 years	66	83.5
**Employed**		
Yes	63	79.7
No	16	20.3
**Relationship status**		
In a relationship	39	49.4
Currently single	40	50.6
**Living alone**		
Yes	38	48.1
No	41	51.9
**Own children**		
Yes	17	21.5
No	62	78.5
**Self-reported exclusiveness (Interest is …)**		
… exclusively in children	11	13.9
… not exclusively in children	67	84.8
… not specified	1	1.3
**Self-reported age group attracted to**		
Prepubertal (pedophile)	1	1.3
Pubertal (hebephile)	3	3.8
Prepubertal and pubertal (pedophile and hebephile)	8	10.1
Prepubertal and adult (pedophile and teleiophile)	4	5.1
Pubertal and adult (hebephile and teleiophile)	27	34.2
Prepubertal, pubertal and adult (pedophile, hebephile, and teleiophile)	35	44.3
Not specified	1	1.3
**Self-reported sexual orientation**		
Attracted to males	14	17.7
Attracted to females	48	60.8
Attracted to both sexes	16	20.3
Not specified	1	1.3
**Self-reported prior lifetime sexual offenses^c^**		
Non-offending	6	7.6
Child sexual abuse only	7	8.9
Child pornography use only	43	54.4
Mixed offenses	23	29.1
**Previously known to justice^c^**		
Child pornography offenses	11	13.9
Child sexual abuse offenses	5	6.3
Child pornography and child sexual abuse offenses	2	2.5
Not previously known to justice	61	77.2

*^a^Absolute share in the sample.*

*^b^Percentage share in the sample.*

*^c^Status when entering the treatment program.*

• not (yet) having offended and/or never having consumed child sexual abuse images, though fearing doing so, or
• already having offended and/or having consumed child sexual abuse images, but not being known to the legal system, or
• previously having been charged with and/or found guilty of relevant offenses and having fully served any sentence received as a result, and fearing committing further offenses(Netzwerk “Kein Täter werden”, 2018).

Treatment involved 90 min of group therapy led by two group therapists weekly, or individual therapy sessions every 1 or 2 weeks. Every individual had to participate in an initial diagnostic procedure that comprised diagnostic interviews, a risk assessment, and a battery of self-report questionnaires. Hereafter, every participant was introduced to the therapeutic team by the therapist who conducted the initial diagnostic procedure. Referral for group vs. individual therapy was debated and decided within the whole team, i.e., medical doctors and psychologists [see Lampalzer et al. (2020) for more detailed information on indicators for group vs. individual treatment in this sample].

The treatment program at the Institute for Sex Research, Sexual Medicine and Forensic Psychiatry is based on the risk-need-responsivity model (Andrews et al., 1990) since, in addition to reducing possible distress from SIM, its main objective is to prevent sexual abuse of children and the use of abusive images. The risk principle determines therapy intensity. According to the need principle therapy focuses on the three most important dynamic risk factors that are related to the individual’s modifiable risk of (re-)offending, e.g., in the realm of intimacy deficits or poor self-regulation. With regard to the responsivity principle referral to group vs. individual treatment, therapeutic technique, and decision for psychiatric treatment or medication in addition to psychotherapy are considered important. In the initial phase of treatment motivation and aims are clarified and biography work is done. In the intermediate phase risk factors and behavioral change are focused, particularly sexual self-regulation, emotional congruence with children, awareness and handling of risk situations, abuse related attitudes, hypersexuality and sexual urges, increase in interpersonal abilities, improvement of coping strategies, and empathy. The final phase concentrates on preventing relapses, considering support groups, and developing future plans (Briken et al., 2018).

After their last treatment session participants normally, i.e., if they were willing to do so, underwent a final diagnostic procedure which consisted, except for some updates, of the same questionnaires as the initial diagnostic procedure.

Five participants, who were included in the present study, had not filled in the Inventory of the Acceptance of Sexual Inclination (IASI rev, Mundt et al., 2011). For this reason, they were excluded from the analysis. The final sample consisted of 79 participants. Their age ranged from 19 to 61 years (*M* = 35.99, *SD* = 11.25). One participant did not indicate his age. Twenty-four (30%) participants were still in treatment, and 55 (70%) had partly or fully completed the treatment program. Of these participants who had partly or fully completed the treatment program 35 (64%) had completed the final diagnostic procedure, including IASI rev. Only these 35 men could be included into pre-post comparison analysis. Treatment duration ranged from 7 to 67 months (*M* = 30.66, *SD* = 15.39).

### Measures

#### Inventory of the Acceptance of Sexual Inclination

The IASI rev is an unpublished self-report questionnaire designed to assess the extent of acceptance or integration of a sexual inclination into the individual self-concept (Ahlers et al., 2008). Sexual inclination is understood as the third of three axes of sexual preference^2^ : “our *sexual inclination* toward a (i) preferred specific *type* of sexual partner and (ii) a preferred specific *mode* of sexual activity” (Schaefer and Ahlers, 2018, p. 88). According to Schaefer and Ahlers, it “resembles the current definition of paraphilias in the DSM-5” which refers to erotic activities (modes) such as spanking and whipping, and erotic targets (types), such as children, corpses or inanimate objects (Schaefer and Ahlers, 2018, p. 89). The IASI rev is a short version of the Inventory of the Acceptance of Sexual Preference (“Inventar zur Akzeptanz der sexuellen Präferenz”; cf. Ahlers et al., 2008) which has four subscales: *Attitude* (subjective attitude toward the acceptance of one’s own sexual preference, extent to which the attitude can become relevant for behavior), *Perceived Acceptance* (extent of real acceptance), *Emotion* (emotional processing of one’s own sexual preference), and *Fantasy and Control* (handling fantasies and needs that correspond to the sexual preference). The IASI rev has 15 items that are answered on a 5-point scale (e.g., “My sexual fantasies scare me.”; “I am aware of my sexual inclination.”) (see Appendix 1 for an English translation including all items). Total scores range from 15 to 75, with higher values indicating a greater acceptance. The IASI rev has not been validated yet. For the present pilot study the IASI rev was found to be highly reliable (15 items; Cronbach’s α = 0.88). The items of the IASI rev do not specifically refer to SIM. However, in the context of the battery of questionnaires that was filled in by the participants of the present study, it is rather unlikely that sexual inclination was not interpreted as SIM. Because the battery is explicitly designated for individuals with SIM and all of the participants turned to the network site specifically due to their SIM. In this study, we used the IASI rev total score for analyzing “acceptance” as a multidimensional construct and Item 5 of IASI rev (“My inclination is an integral part of my personality.”) for focusing even more on the aspect of (im-)mutability of SIM. This item of IASI rev (hereinafter also referred to as “Integral Part Item”) most closely corresponds to this aspect.

#### STABLE-2007

The STABLE-2007 (Hanson et al., 2007; Matthes and Rettenberger, 2008) is a 13-item risk assessment tool to measure dynamic risk for contact sexual recidivism among adult males who have been charged with a sexual offense. The 13 risk factors are evaluated by third party. They have been shown to be associated with sexual recidivism and are systematized into five sections: *Significant Social Influences*, *Intimacy Deficits* (capacity for relationship stability, emotional identification with children, hostility toward women, general social rejection, lack of concern for others), *General Self-Regulation* (impulsivity, poor problem-solving skills, negative emotionality), *Sexual Self-Regulation* (sex drive and preoccupations, sex as coping, deviant sexual preference), and *Cooperation with Supervision*. All of the items are scored on a 3-point scale. Total scores range from 0 to 26 (exception: emotional identification is not scored for offenders not having a child as a victim so that for them total scores only range to 24), with higher values indicating a higher dynamic risk of recidivism. The STABLE-2007 has proven very good interrater reliability for the English version [ICC = 0.79 (Hanson et al., 2007)] and for the German version [ICC = 0.90 for a population of sex offenders with 50.9% being child molesters (Eher et al., 2012); ICC = 0.90 for a population of child molesters (Rettenberger et al., 2011)], too. It has also demonstrated good predictive validity for recidivism [AUC = 0.67–0.71 for sexual, violent and general recidivism (Eher et al., 2012)]. As mentioned above, the STABLE-2007 is designed for contact sexual offenders who have been charged with a sexual offense. The dynamic risk factors of the STABLE-2007 are not validated for recidivism relating to child/adolescent sexual abuse imagery and not for individuals with SIM without or with undetected offences. Nevertheless, the STABLE-2007 is used in this exploratory pilot study because it measures stable dynamic risk factors and there is no established tool available yet for this specific group with SIM. However, data are analyzed for the group of patients in this sample who reported child/adolescent sexual abuse in their past as well as for the whole group. Because one of the tool’s developers, Karl Hanson, explicitly recommended to not use it for internet offenders who only offended with indecent images of children, which might apply to the majority of the sample, and not with identifiable victims (Webb, 2018, p. 107).

#### Items Assessing the Frequency of the Use of Child Abuse/Exploitation Material and Frequency of Sexual Desire/Behavior Toward Children

A subset of items of questionnaires of the initial diagnostic procedure which assess the frequency of consumption of (sexual) imagery of children, adolescents, adolescents sexually interacting with children, adults sexually interacting with children, and adults sexually interacting with adolescents is used, as well as a subset of items assessing the frequency of desire for sexual activities, and actual sexual activities with minors. In order to reduce the number of these items and generate six adequate items for the analysis, we amalgamated the relevant items via taking the score of the highest frequency of the relevant items. The six items are (see Appendix 2 for the specific questions of three different batteries):

(1) Frequency of Use of Legal Imagery of Children;
(2) Frequency of Use of Legal Imagery of Adolescents;
(3) Frequency of Use of Illegal Child Sexual Abuse Imagery;
(4) Frequency of Use of Illegal Adolescent Sexual Abuse Imagery;
(5) Frequency of Desire for Sexual Activities with Minors;
(6) Frequency of Sexual Activities with Minors.

Via these six items common distinctions in the literature are represented: (1) legal vs. illegal imagery, (2) pedophilic interests vs. hebephilic interests, (3) use of child abuse/exploitation material (hands-off) vs. (drive to) child sexual abuse (hands-on). Frequency is rated on a 5-point-Likert scale, with the following answer options: 1 = “never,” 2 = “few times,” 3 = “monthly,” 4 = “weekly,” 5 = “daily.” For amalgamation, the score of the highest frequency of the relevant items was taken because this corresponds to the answer the participant would have given if he had answered to the amalgamated item. The distinction between legal and illegal imagery follows the COPINE scale (Quayle, 2008), with category 1–3 classified as legal imagery and category 4–10 as illegal imagery. Table 2 shows that inter-item correlations were significant and between 0.23 and 0.68, except for item 6 that was not significantly correlated to the items 2, 3, and 4.

**TABLE 2:** Results of Pearson correlation tests for inter-item correlations of the items for frequency of the use of child abuse/exploitation material and frequency of sexual desire/behavior toward children inter-item correlation.

Items	1	2	3	4	5	6
(1) Use of Legal Imagery of Children	1					
(2) Use of Legal Imagery of Adolescents	0.53**	1				
(3) Use of Illegal Child Sexual Abuse Imagery	0.66**	0.42**	1			
(4) Use of Illegal Adolescent Sexual Abuse Imagery	0.33**	0.68**	0.62**	1		
(5) Desire for Sexual Activities with Minors	0.45**	0.26*	0.45**	0.29*	1	
(6) Sexual Activities with Minors	0.23**	−0.06	0.10	−0.12	0.32**	1

*Correlation is significant at the 0.05 level.

**Correlation is significant at the 0.01 level.

### Statistical Analysis

As the battery of questionnaires was revised with regard to the current state of research in the course of data collection, not all questionnaires used in the present study were filled in by all participants. As the STABLE-2007 was only added to the battery of questionnaires in 2014, it was filled in for many participants by the therapists at a later time during the course treatment. Only those participants (*n* = 45) were included in the statistical analysis of correlations regarding the STABLE-2007 for whom the STABLE-2007 was completed not more than 6 months after the participant had completed the self-report questionnaires of the battery, assuming that no substantial change in dynamic risk of sexual recidivism has taken place during this time. In a second analysis of correlations regarding the STABLE-2007, only those (*n* = 16) were included who reported child/adolescent sexual abuse in their past (see section “STABLE-2007” for explanation).

First, the relation between acceptance of SIM and dynamic risk for contact sexual recidivism before treatment, i.e., between IASI rev total score/Item 5 IASI rev score and STABLE-2007 total score of the initial diagnostic procedure, was analyzed using the Spearman’s correlation coefficient because variables were ordinally scaled (Upton and Cook, 2014). Second, the relation between acceptance of SIM and frequency/intensity of the use of material of abuse/exploitation of minors, i.e., IASI rev total score/Item 5 IASI rev score and the items of the initial diagnostic procedure assessing the frequency of the use of child abuse/exploitation material, was analyzed using the Spearman’s correlation coefficient because variables were ordinally scaled, too (Upton and Cook, 2014). Third, a Spearman’s correlation was run to determine the relationship between acceptance of SIM and frequency/intensity of sexual desire/behavior toward minors, i.e., IASI rev total score/Item 5 IASI rev score and items assessing the frequency of sexual desire/behavior toward children, because variables were also ordinally scaled (Upton and Cook, 2014). Fourth, a Wilcoxon signed-rank-test was performed to compare acceptance of SIM, i.e., IASI rev total score, between initial and final diagnostic procedure because the data were not normally distributed. Fifth, a paired-samples *t*-test was carried out to compare Item 5 IASI rev score between initial and final diagnostic procedure because the data were normally distributed (Kim, 2015). In the final diagnostic procedure, the IASI rev was completed by 35 participants. Significance was set at a value less than 0.05. All statistical analyses were conducted using SPSS (V 24) (IBM SPSS Statistics, IBM Corporation, Armonk, NY, United States).

## Results

### Relationship Between Inventory of the Acceptance of Sexual Inclination and STABLE-2007

For the whole group, results of the Spearman’s correlation indicated that there was no significant correlation between IASI rev total score and STABLE-2007 total score (*r*_*s*_ = –0.22, *n* = 43, *p* = 0.166), and that there was no significant correlation between Item 5 IASI rev score and STABLE-2007 total score (*r*_*s*_ = 0.07, *n* = 45, *p* = 0.642) (Table 3).

**TABLE 3:** Descriptive statistics and correlations between IASI rev (Item 5 IASI rev) and STABLE-2007, Frequency of Use of Legal Imagery of Children, Frequency of Use of Legal Imagery of Adolescents, Frequency of Use of Illegal Child Sexual Abuse Imagery, Frequency of Use of Illegal Adolescent Sexual Abuse Imagery, Frequency of Desire for Sexual Activities with Minors, and Frequency of Sexual Activities with Minors.

Variable		*M*	*SD*	*Mdn*	*Range*	*r* ^a^	*p* ^a^	FDR adjusted *p*_BH_^a,b^	*n* ^a^
(1) IASI rev		51.20	11.03	51	28–73	–	–		–
	(a) Item 5 IASI rev	3.30	1.21	3	1–5	–	–		–
(2) STABLE-2007									
	(a) Total sample	9.49	2.94	9	4–16	–0.215 0.071	0.166 0.642	0.266 0.685	43 45
	(b) Participants with child sexual abuse in the past	10.75	2.72	11	5–15	0.013 –0.178	0.961 0.509	0.961 0.582	16 16
(3) Frequency of Use of Legal Imagery of Children		2.75	1.53	3	1–5	0.413* 0.323*	<0.001 0.005	<0.001 0.040	72 75
(4) Frequency of Use of Legal Imagery of Adolescents		2.35	1.43	2	1–5	0.224 0.084	0.058 0.474	0.133 0.582	72 75
(5) Frequency of Use of Illegal Child Sexual Abuse Imagery		2.86	1.46	3	1–5	0.265 0.183	0.026 0.119	0.083 0.213	70 74
(6) Frequency of Use of Illegal Adolescent Sexual Abuse Imagery		2.68	1.42	3	1–5	0.109 –0.111	0.367 0.344	0.489 0.489	71 75
(7) Frequency of Desire for Sexual Activities with Minors		3.09	1.46	4	1–5	0.234 0.176	0.044 0.120	0.117 0.213	75 79
(8) Frequency of Sexual Activities with Minors		1.57	1.17	1	1–5	0.304* 0.264	0.008 0.019	0.043 0.076	75 79

*p_BH_ < 0.05.

*^a^The first line refers to IASI rev, the second line refers to Item 5 of IASI rev.*

*^b^Adjusted p-value using the Benjamini–Hochberg procedure (Benjamini and Hochberg, 1995; Hemmerich, 2016).*

*IASI, Inventory of the Acceptance of Sexual Inclination.*

Similarly, for the group of patients who reported child/adolescent sexual abuse in their past, results of the Spearman’s correlation indicated that there was no significant correlation between IASI rev total score and STABLE-2007 total score (*r*_*s*_ = 0.01, *n* = 16, *p* = 0.961), and no significant correlation between Item 5 IASI rev score and STABLE-2007 total score (*r*_*s*_ = –0.18, *n* = 16, *p* = 0.509), either (Table 3).

### Relationship Between Inventory of the Acceptance of Sexual Inclination and Frequency/Intensity of the Use of Material of Abuse/Exploitation of Minors

Results of the Spearman’s correlation indicated that there was a medium^3^, positive correlation between IASI rev total score and Frequency of Use of Legal Imagery of Children score (*r*_*s*_ = 0.41, *n* = 72, *p* ≤ 0.001), and no significant correlation between IASI rev total score and Frequency of Use of Legal Imagery of Adolescents score (*r*_*s*_ = 0.22, *n* = 72, *p* = 0.058). There was also a medium, positive correlation between Item 5 IASI rev score and Frequency of Use of Legal Imagery of Children score (*r*_*s*_ = 0.32, *n* = 75, *p* = 0.005), and no significant correlation between Item 5 IASI rev score and Frequency of Use of Legal Imagery of Adolescents score (*r*_*s*_ = 0.08, *n* = 75, *p* = 0.474), either. The correlations remained statistically significant after Benjamini–Hochberg correction (Table 3).

Moreover, results of the Spearman’s correlation indicated that there was a small, positive correlation between IASI rev total score and Frequency of Use of Illegal Child Sexual Abuse Imagery score (*r*_*s*_ = 0.27, *n* = 70, *p* = 0.026), no significant correlation between IASI rev total score and Frequency of Use of Illegal Adolescent Sexual Abuse Imagery score (*r*_*s*_ = 0.11, *n* = 71, *p* = 0.367), no significant correlation between Item 5 IASI rev score and Frequency of Use of Illegal Child Sexual Abuse Imagery score (*r*_*s*_ = 0.18, *n* = 74, *p* = 0.119), and no significant correlation between Item 5 IASI rev score and Frequency of Use of Illegal Adolescent Sexual Abuse Imagery score (*r*_*s*_ = –0.11, *n* = 75, *p* = 0.344). The correlation between IASI rev total score and Frequency of Use of Illegal Child Sexual Abuse Imagery score was not statistically significant after Benjamini–Hochberg correction (Table 3).

### Relationship Between Inventory of the Acceptance of Sexual Inclination and Frequency/Intensity of Sexual Tendencies/Behavior Toward Minors

Results of the Spearman’s correlation indicated that there was a small, positive correlation between IASI rev total score and Frequency of Desire for Sexual Activities with Minors score (*r*_*s*_ = 0.23, *n* = 75, *p* = 0.044). Results of the Spearman correlation also indicated that there was a medium, positive correlation between IASI rev total score and Frequency of Sexual Activities with Minors score (*r_*s*_* = 0.30, *n* = 75, *p* = 0.008). There was no significant correlation between Item 5 IASI rev score and Frequency of Desire for Sexual Activities with Minors score (*r*_*s*_ = 0.18, *n* = 79, *p* = 0.120), and a small, positive correlation between Item 5 IASI rev score and Frequency of Sexual Activities with Minors score (*r*_*s*_ = 0.26, *n* = 79, *p* = 0.019). After Benjamini–Hochberg correction, only the correlation between IASI rev total score and Frequency of Sexual Activities with Minors score remained statistically significant (Table 3).

### Pre–post Comparison of Inventory of the Acceptance of Sexual Inclination

Results of the Wilcoxon signed-rank tests indicated no statistical difference between IASI rev total score before (*Mdn* = 54) and after (partial) completion of treatment (*Mdn* = 55), *T* = 261, *z* = –0.26, *p* = 0.799, *r* = –0.03 (Table 4). A paired-samples t-test indicated no statistical difference between the score of Item 5 of IASI rev before beginning treatment (*M* = 3.51, *SD* = 1.07) and the score of Item 5 of IASI rev after (partial) completion of treatment (*M* = 3.57, *SD* = 1.12), *t*(34) = –0.26, *p* = 0.797, *d* = –0.05, either (Table 4).

**TABLE 4:** Results of Wilcoxon signed-rank test and paired-samples *t*-test and descriptive statistics for IASI rev total score and Item 5 IASI rev score (*n* = 35).

Outcome	Pre-test					Post-test					95% CI for mean difference						
	*M*	*SD*	*Mdn*	*Range*	*n*	*M*	*SD*	*Mdn*	*Range*	*n*		*Z*	*t*	*p*	FDR adjusted *p*_BH_^a^	*Effect size r*	*Effect size d*
IASI rev	52.50	10.44	54	28–73	34	52.43	10.32	55	25–69	35		–0.26		0.799	0.799	–0.03	
Item 5 IASI rev	3.51	1.07	4	1–5	35	3.57	1.12	4	1–5	35	–0.51, 0.39		–0.26	0.797	0.799		–0.05

*p_BH_ < 0.05.

*^a^Adjusted p-value using the Benjamini–Hochberg procedure (Benjamini and Hochberg, 1995; Hemmerich, 2016).*

*IASI, Inventory of the Acceptance of Sexual Inclination.*

## Discussion

### General Discussion

This exploratory pilot study investigated if acceptance of SIM is associated with pedophilia associated urges and behaviors. Acceptance of SIM as measured by IASI rev total score and the score of the item “My inclination is an integral part of my personality” were not related to dynamic risk factors for contact sexual reoffending as measured by STABLE-2007. It is possible that this is due to the sample which might be characterized by other dynamic risk factors (77% were not previously known to justice) than forensic samples of contact sexual offenders with a SIM who have been charged with a sexual offense. Maybe the STABLE-2007 is not an adequate instrument for measuring dynamic risk for contact sexual reoffending in this sample. However, there was no correlation between IASI rev total score or Integral Part Item score and STABLE-2007 score, either. This might also be due to a lack of statistical power for finding small effects. Power analyses using G*Power (Faul et al., 2007) indicated that, with 80% power and α = 0.05, a sample size of 129 would be required to detect an effect of *r* = 0.215 that was revealed for the whole sample in the present study, and a sample size of 1,222 for an effect of *r* = 0.071 that was revealed for the subsample with child sexual abuse in the past.

Results of the current exploratory pilot study indicate a medium, positive correlation between acceptance of SIM and the frequency of the use of legal imagery of children, and a small, positive correlation between acceptance of SIM and the frequency of the use of illegal child sexual abuse imagery. This means, the more participants report to accept their SIM, the more they also report to use legal imagery of children as well as illegal child sexual abuse imagery, or vice versa. However, the findings reveal no correlation between acceptance of SIM and the frequency of use of legal imagery of adolescents, and no correlation between acceptance of SIM and the frequency of the use of illegal adolescent sexual abuse imagery. Furthermore, they show a small, positive correlation between acceptance of SIM and the reported frequency of desire for sexual activities with minors, and a medium positive correlation between acceptance of SIM and the reported frequency of sexual activities with minors.

The results may suggest that more acceptance of SIM might be associated with more awareness of a SIM, the desire might be more present and associated with more use of legal imagery and even more proneness to sexual abuse. Less acceptance of SIM, however, might be associated with more motivation to work on diminishing pedophilia associated urges and behaviors and thus make individuals reduce the focus on desire for sexual activities with children, use less legal and illegal material and prevent themselves from committing child sexual abuse. Of course, as we solely conducted correlation analyses, we are not able to make any statement about causality.

It is surprising that the correlations that were found for legal and illegal imagery of children were not found for legal and illegal imagery of adolescents. Research suggests that pedophilic, hebephilic and teleiophilic individuals are different from each other, but there is still a lack of research in this field (Sea and Beauregard, 2018). In the present study, the majority of participants (44%) were pedophilic, hebephilic and teleiophilic, or hebephilic and teleiophilic (34%) at the same time. Obviously, these sexual interests cannot be seen as totally distinct from each other. According to the findings of this exploratory pilot study, acceptance of SIM might be different in the context of a pedophilic interest than in the context of a hebephilic interest. Maybe acceptance of a pedophilic interest has a deeper impact on awareness and the frequency of corresponding sexual desire and sexual behavior than acceptance of a hebephilic interest (cf. Prentky and Barbaree, 2011; Calkins Mercado and Beattey, 2012).

Additionally, results of the current exploratory pilot study indicate a medium, positive correlation between the assumption of SIM as an integral part of one’s personality and the frequency of the use of legal imagery of children, and a small, positive correlation between this assumption and the frequency of sexual activities with minors, but no correlation between this assumption and any of the other frequency measures used. This means, the more men regard their pedophilic interests as an integral part of their personality, the more they sexualize non-sexual representations of children and commit hands-on child sexual abuse, or vice versa, the more men sexualize non-sexual representations of children and commit hands-on child sexual abuse, the more they regard their pedophilic interests as an integral part of their personality.

It has to be noted that after correction for multiple testing, only the correlation between acceptance of SIM and the frequency of the use of legal imagery of children, the correlation between the assumption of SIM as an integral part of one’s personality and the frequency of the use of legal child sexual abuse imagery, and the correlation between acceptance of SIM and the frequency of sexual activities with minors remained statistically significant.

*Post hoc* power analyses that were conducted using the program G*Power (Faul et al., 2007) indicated that the statistical power for the correlation analyses of this study (assuming *n* = 70; see Table 2) was 13% for detecting a small effect of *r* = 0.1, 80% for detecting a medium effect of *r* = 0.32 and more than 99% for detecting a large effect of *r* = 0.5 (according to Cohen, 1988, 1992), with α = 0.05. Thus, there was more than sufficient power (i.e., 80%) at the large effect size level, quite enough power at the medium effect size level and less than sufficient statistical power at the effect size level of less than *r* = 0.32.

The present study also investigated whether acceptance of SIM changed in the course of treatment. Results indicate no difference between before beginning treatment and (partial) completion of treatment. Maybe for a change of acceptance of SIM it would have to be directly targeted in treatment. However, treatment has a particular focus on risk factors (cf. Institute for Sexology and Sexual Medicine of the Charité, 2013; Briken et al., 2018; Netzwerk “Kein Täter werden”, 2018) and acceptance of SIM might not have appeared to be a relevant risk factor. Another reason why acceptance of SIM did not change might be that the period under study was not sufficiently long enough to observe a change in acceptance of SIM. Furthermore, insufficient statistical power because of the small sample size in the present study (*n* = 35) may have played a role in limiting the significance of the pre-post comparison conducted. Effect sizes from a study by Engel et al. (2018) with a sample of the Prevention network “Kein Täter werden” were between *d* = –0.14 and *d* = –0.58 for comparisons of treatment group (*n* = 35) before and after therapy regarding several measures. This supports the expectation for medium effects (according to Cohen, 1988, 1992). For the present study, a power analysis using the program G*Power (Faul et al., 2007) indicated that a total sample of 35 participants would be needed to detect a medium effect (*d* = 0.5) with 80% power and α = 0.05. This is equivalent to the sample size of the present study.

### Limitations and Future Studies

The generalizability of the present results is restricted due to the sample size of only 79 participants in the whole sample and only 35 participants in the sample for pre-post comparison. With this sample size, especially with regard to pre-post comparison, this exploratory pilot study was underpowered for small effects. Therefore, the results of the present study need to be replicated with studies that include larger samples that would guarantee a sufficient statistical power. Generalizability is also limited because of the particular characteristics and institutional context of the *“*Kein Täter Werden” network site in Hamburg. Furthermore, 13 men could not be included in the study because they had not provided informed consent. Therefore, these data, and maybe specific characteristics, are not represented in the findings of this study.

Concerning validity, our results are limited because almost only self-report measurements with forced-choice categories were used. Forced-choice categories may simplify answers and/or distort information because of the particular choice sets given. The patients’ self-report was not validated by objective measures. Hence, it cannot be ruled out that an effect of social desirability distorted our data. Only the STABLE-2007 items are assessed by third party. For a part of the patients whose STABLE-2007 date were analyzed the STABLE-2007 was only filled in for a period of a few weeks (for 25 patients more than 6 weeks later) up to 6 months after completion of the initial diagnostic procedure, because the STABLE-2007 was not part of the battery of questionnaires right from the beginning of data collection. It cannot be ruled out that this retrospective completion may have been biased. However, as mentioned above, we assume that no substantial change in dynamic risk of sexual recidivism takes place during this time. Moreover, the self-report questionnaires analyzed in this exploratory pilot study are not validated, yet. Thus, as mentioned before, the findings can only be seen as preliminary results.

Until now, acceptance of SIM and its effects are not well researched, yet. There is a need for a validation study of the IASI rev. Furthermore, at this state of research, case studies and qualitative research would help to understand this construct and its impact on individuals better (see Jones et al., 2020 for qualitative research giving some insights into acceptance of pedophilia as a coping strategy of individuals who identify as pedophilic or hebephilic and do not offend). Quantitative research is needed in order to study how closely acceptance of SIM is related to measures of other constructs like the Unconditional Self-Acceptance Questionnaire (Chamberlain and Haaga, 2001), the Emotional Processing Scale (Baker et al., 2015), and/or the Thought Control Questionnaire (Wells and Davies, 1994). For a broader treatment plan, going beyond directly focusing on the main risk factors, research on the relation of acceptance of SIM to constructs associated with emotional well-being should be conducted, e.g., on how closely it is related to the context of personal suffering, including ambivalent identity experience (Blagden et al., 2018) and stigma-related stress (Jahnke et al., 2015; Wagner et al., 2016; Lievesley et al., 2020; Stelzmann et al., 2020).

Since we solely calculated correlation coefficients, we are not able to make any statement about causal relations. This means, it is not clear whether the acceptance of SIM had an impact on the other variables, or vice versa, or whether and how they interact with each other. A further study should include statistical analyses which allow the examination of causality (e.g., crossed-lagged panel analyses; Frees, 2004).

The present study focused on the question if acceptance of SIM is related to essential treatment outcomes. The outcome measures of the present study, except of “Frequency of Desire for Sexual Activities with Minors,” mainly focus on the frequency of behavior. Further studies could also include outcome measures that focus on the strength of SIM in itself (see Carvalho et al., 2020 for different measures that are available), thus highlighting that not only behavioral changes are notable treatment goals but also change of SIM in itself, in general or via a partial shift from pedophilic to teleiophilic interests, for example. Accordingly, it would be worth studying if the level of SIM is associated with acceptance of SIM.

With larger samples, in the next stage of research, subgroups of men with SIM should be studied in more detail with regard to acceptance of SIM in order to be able to understand their characteristics and personalities better, differentiate better between them, and adapt treatment accordingly. There might be differences between the subgroups of non-offenders, mixed offenders, hands-on sexual offenders, and offenders without hands-on offenses but consumption of material depicting the sexual exploitation of minors. These subgroups were not differentiated in this study. Previous research indicates that these subgroups are, among other things, distinctive from each other regarding indicators of antisociality (Babchishin et al., 2015), criminal history (Long et al., 2013), offense supportive attitudes (Jahnke et al., 2015), stability of life factors, and substance abuse problems (Ly et al., 2018). Maybe they also differ from each other regarding acceptance of SIM. Previous findings suggest that offenders without hands-on offenses but consumption of material depicting the sexual exploitation of minors have greater levels of SIM than hands-on sexual offenders in the sense of greater sexual deviancy (even if going along with greater barriers to hands-on offending), greater likelihood to have problems with sexual preoccupation and sexual self-regulation (Babchishin et al., 2015), and a stronger diagnostic indicator of pedophilia (Seto et al., 2006). Another subgroup worth studying are webcam child sexual abuse offenders (de Tribolet-Hardy et al., 2020).

Differences in acceptance of SIM between individuals who are attracted to children, individuals who are attracted to adolescents, those who are attracted to both, and those who are also attracted to adults also need to be investigated in greater detail. Last but not least, prior research shows that social factors, such as social support and relationship status, affect self-acceptance (Leavy and Adams, 1986; Vincke and Bolton, 1994; Huang et al., 2020). Thus, they might as well affect acceptance of SIM. Therefore, further research should also examine relationships between social factors, e.g., relationship status and living alone vs. not living alone, acceptance of SIM and the effects on treatment outcomes.

## Conclusion

The main question of this study was if acceptance of SIM is related to essential treatment outcomes. The findings, on the one hand, indicate a positive correlation between acceptance of SIM and the use of legal imagery of children. On the other hand, they suggest that acceptance of SIM might be positively correlated with illegal activities, such as the frequency of sexual activities with minors. According to this, more acceptance might reinforce or be reinforced by legal ways of dealing with pedophilic interest, but it might also pave the way for or be increased by an enhancement of illegal activities. Hence, acceptance of SIM should be further investigated before specific recommendations for treatment are made. With our current knowledge, there is a need for individualized treatment plans allowing for a modification of SIM in some patients and, in other patients, for working on acceptance of SIM.

## Data Availability Statement

The datasets for this study are not publicly available due to patient confidentiality and participant privacy. The computer code is available on request to the corresponding author.

## Ethics Statement

The studies involving human participants were reviewed and approved by Ethics Committee of the Chamber of Psychotherapists Hamburg (09/2019-PTK-HH, 02/2015-PTK-HH). The patients/participants provided their written informed consent to participate in this study.

## Author Contributions

Under the supervision of PB, UL conceptualized the study and was the primary writer of the manuscript. UL and ST prepared the data for analysis. All the authors had full access to all the data in the study and take responsibility for the integrity of the data and the accuracy of data analysis, were involved in developing, editing, reviewing, and providing feedback for this manuscript, and have given approval of the final version to be published.

## Conflict of Interest

The authors declare that the research was conducted in the absence of any commercial or financial relationships that could be construed as a potential conflict of interest.

## Publisher’s Note

All claims expressed in this article are solely those of the authors and do not necessarily represent those of their affiliated organizations, or those of the publisher, the editors and the reviewers. Any product that may be evaluated in this article, or claim that may be made by its manufacturer, is not guaranteed or endorsed by the publisher.

## Footnotes

1 The findings mentioned were criticized as insufficient because of still unclear definitions (e.g., defining pedohebephilic interests as a SIM who are at least five years younger than oneself vs. defining pedohebephilic interest on the basis of a developmental approach involving biological, social, psychological, and experiential aspects that started before experiencing a SIM for the first time), not enough consideration of developmental processes (e.g., transitional processes, the fact that age of onset is maybe a part of ongoing developmental processes instead of a discrete event) and not sufficiently precise operationalization of age of onset (i.e., asking about age of first SIM might be misunderstood because almost every person is attracted to minors at some point in life) (McPhail, 2018).

2 The other two axes are: “(a) our *sexual orientation* towards the preferred sex of the desired sexual partner […], (b) our *sexual alignment* towards the preferred stage of physical development of the sexual partner’s body” (Schaefer and Ahlers, 2018, p. 88).

3 The Spearman’s correlation coefficients are interpreted according to Cohen (1988, 1992).

## Appendixes

**APPENDIX 1:** IASI rev (Mundt et al., 2011).

Instruction: In the following you are asked to give some information on how you handle your sexual inclination.					
**This statement is true…**	**Not at all**	**Little**	**Moderately**	**Fairly**	**Very much**

(1) I forbid myself my sexual fantasies.	1	2	3	4	5
(2) I can enjoy my sexual fantasies without a bad conscience.	1	2	3	4	5
(3) I hate my sexual inclination.	1	2	3	4	5
(4) I cannot accept my sexual inclination.	1	2	3	4	5
(5) My inclination is an integral part of my personality.	1	2	3	4	5
(6) I reject my sexual inclination.	1	2	3	4	5
(7) One must resist disagreeable sexual fantasies.	1	2	3	4	5
(8) I allow myself my sexual fantasies.	1	2	3	4	5
(9) I reject myself because I have this sexual inclination.	1	2	3	4	5
(10) My sexual fantasies have nothing to do with me.	1	2	3	4	5
(11) My sexual fantasies scare me.	1	2	3	4	5
(12) I have no sexual fantasies.	1	2	3	4	5
(13) I am aware of my sexual inclination.	1	2	3	4	5
(14) I like to embellish my sexual fantasies.	1	2	3	4	5
(15) I resist my sexual fantasies.	1	2	3	4	5

**APPENDIX 2:** The specific descriptions/questions that the six items represent which assess the frequency of the use of child abuse/exploitation material and frequency of sexual desire/behavior towards children.

(1) Frequency of Use of Legal Imagery of Children	• How often in the last 6 months did you consume (1) films with, (2) images/photos of lightly dressed children (e.g., in underwear, gym shorts, swimming trunks, leotards, transparent clothing…) for (a) masturbation, (b) stimulating pastime? • How often in the last 6 months did you consume films with normally dressed children (e.g., children’s films, feature films, documentary films…) for (a) masturbation, (b) stimulating pastime? • How often in the last 6 months did you consume images/photos/portraits of dressed children (e.g., in magazines, on postcards, in illustrated books…) for (a) masturbation, (b) stimulating pastime? • How often in the last 6 months did you consume imagery of (a) prepubescent, (b) early pubescent children – dressed in non-sexual representations – for masturbation or stimulating pastime? • How often in the last 6 months did you consume imagery of (a) prepubescent, (b) early pubescent children – lightly dressed (e.g., in underwear) or naked in non-sexual representations – for masturbation and/or stimulating pastime? • In the last 12 months I used media for sexual arousal which depict a child under 14 years of age with a (a) prepubescent, (b) early pubescent, (c) late pubescent body scheme, dressed, lightly dressed (e.g., in underwear) or naked in real non-sexual representations (or natural poses, respectively).
(2) Frequency of Use of Legal Imagery of Adolescents	• How often in the last 6 months did you consume (1) films with, (2) images/photos of lightly dressed adolescents (e.g., in underwear, gym shorts, swimming trunks, leotards, transparent clothing…) for (a) masturbation, (b) stimulating pastime? • How often in the last 6 months did you consume films with normally dressed adolescents (e.g., children’s films, feature films, documentary films…) for (a) masturbation, (b) stimulating pastime? • How often in the last 6 months did you consume images/photos/portraits of dressed adolescents (e.g., in magazines, on postcards, in illustrated books…) for (a) masturbation, (b) stimulating pastime? • How often in the last 6 months did you consume imagery of late pubescent adolescents – dressed in non-sexual representations – for masturbation and/or stimulating pastime? • How often in the last 6 months did you consume imagery of late pubescent adolescents – lightly dressed (e.g., in underwear) or naked in non-sexual representations – for masturbation and/or stimulating pastime? • In the last 12 months I used media for sexual arousal which depict an adolescent between 14 and 17 years of age, dressed, lightly dressed (e.g., in underwear) or naked in real non-sexual representations (or natural poses, respectively).
(3) Frequency of Use of Illegal Child Sexual Abuse Imagery	• How often in the last 6 months did you consume (1) films with, (2) images/photos of naked children (e.g., on the beach, bathing, medically examined, or posing in front of a camera) for (a) masturbation, (b) stimulating pastime? • How often in the last 6 months did you consume images/photos of children who were dressed up or posed styled up (e.g., made up, in special clothing, school uniforms, sailor suits etc.) for (a) masturbation, (b) stimulating pastime? • How often in the last 6 months did you consume (1) films, (2) images/photos with children in which sexual organs (e.g., buttocks, penis, vagina, breasts…) can be seen in detail and/or in which a child masturbates for (a) masturbation, (b) stimulating pastime? • How often in the last 6 months did you consume (1) films, (2) images photos with children in/on which sexual acts are performed between an adult and a child for (a) masturbation, (b) stimulating pastime? • How often in the last 6 months did you consume imagery of (a) prepubescent, (b) early pubescent children – dressed, lightly dressed (e.g., in underwear), or naked in erotic/slinky/provocative poses – for masturbation and/or stimulating pastime? • How often in the last 6 months did you consume imagery of (a) prepubescent, (b) early pubescent children which show genital and anal area in detail for masturbation and/or stimulating pastime? • How often in the last 6 months did you consume imagery of (a) prepubescent, (b) early pubescent children which show sexual acts between pre-/early pubescent children (touching, masturbation, mutual stimulation, oral/vaginal/anal intercourse) for masturbation and/or stimulating pastime? • How often in the last 6 months did you consume imagery of (a) prepubescent, (b) early pubescent children which show sexual acts with an adult (touching, masturbation, mutual stimulation, oral/vaginal/anal intercourse) for masturbation and/or stimulating pastime? • In the last 12 months I used media for sexual arousal which depict real children under 14 years of age with a (a) prepubescent, (b) early pubescent, (c) late pubescent body scheme, dressed, lightly dressed (e.g., in underwear) or naked in erotic/slinky/provocative poses. • In the last 12 months I used media for sexual arousal which depict real children under 14 years of age with a (a) prepubescent, (b) early pubescent, (c) late pubescent body scheme and show genital and/or anal area in detail or which show sexual acts without adults being involved. • In the last 12 months I used media for sexual arousal which depict real children under 14 years of age with a (a) prepubescent, (b) early pubescent, (c) late pubescent body scheme and show sexual acts with adults being involved.
(4) Frequency of Use of Illegal Adolescent Sexual Abuse Imagery	• How often in the last 6 months did you consume (1) films with, (2) images/photos of naked adolescents (e.g., on the beach, bathing, medically examined, or posing in front of a camera) for (a) masturbation, (b) stimulating pastime? • How often in the last 6 months did you consume images/photos of adolescents who were dressed up or posed styled up (e.g., made up, in special clothing, school uniforms, sailor suits etc.) for (a) masturbation, (b) stimulating pastime? • How often in the last 6 months did you consume (1) films, (2) images/photos with adolescents in/on which sexual organs (e.g., buttocks, penis, vagina, breasts…) can be seen in detail and/or in which an adolescent masturbates for (a) masturbation, (b) stimulating pastime? • How often in the last 6 months did you consume (1) films, (2) images/photos with children in/on which sexual acts are performed between an adult and an adolescent for (a) masturbation, (b) stimulating pastime? • How often in the last 6 months did you consume imagery of late pubescent adolescents – dressed, lightly dressed (e.g., in underwear), or naked in erotic/slinky/provocative poses for masturbation and/or stimulating pastime? • How often in the last 6 months did you consume imagery of late pubescent adolescents which show genital and anal area in detail for masturbation and/or stimulating pastime? • How often in the last 6 months did you consume imagery of late pubescent adolescents which show sexual acts between late pubescent adolescents (touching, masturbation, mutual stimulation, oral/vaginal/anal intercourse) for masturbation and/or stimulating pastime? • How often in the last 6 months did you consume imagery of late pubescent adolescents which show sexual acts with an adult (touching, masturbation, mutual stimulation, oral/vaginal/anal intercourse) for masturbation and/or stimulating pastime? • In the last 12 months I used media for sexual arousal which depict a real adolescent between 14 and 17 years of age, dressed, lightly dressed (e.g., in underwear) or naked in erotic/slinky/provocative poses. • In the last 12 months I used media for sexual arousal which depict real adolescents between 14 and 17 years of age and show genital and/or anal area in detail or which show sexual acts without adults being involved. • In the last 12 months I used media for sexual arousal which depict real adolescents between 14 and 17 years of age and show sexual acts with adults being involved.
(5) Frequency of Desire for Sexual Activities with Minors	• How often do you have a desire for sex with intercourse with a child/adolescent? • How often do you have a desire for sex without intercourse with a child/adolescent? • How often do you have a desire for intimate body contact with a child/adolescent? • I had sexual desire (= a state of a strong longing or strong desire which arises in thought, triggered by, for example, certain encounters, pictures, moods or feelings; not to be equated with masturbation or other sexual acts) with regard to persons with a (a) prepubescent, (b) early pubescent, (c) late pubescent, (d) postpubescent body scheme, whether or not an act followed or not.
(6) Frequency of Sexual Activities with Minors	• How often do you normally have intercourse with a child/adolescent? • How often do you normally have sex without intercourse with a child/adolescent? • How often do you normally have intimate body contact with a child/adolescent? • In the last 12 months I performed sexual acts without penetration with (a) prepubescent, (b) early pubescent children, (c) late pubescent children under 14 years of age, (d) adolescents between 14 and 17 years of age (e.g., observing/photographing/filming in intimate situations or of charges, peeping, sexualized talk, watching porn together, invitation to (mutual) masturbation, (deep) kiss, masturbation in front of a child/adolescent, rubbing, exposing oneself etc.) • In the last 12 months I performed sexual acts with penetration with (a) prepubescent, (b) early pubescent children, (c) late pubescent children under 14 years of age, (d) adolescents between 14 and 17 years of age (e.g., intercourse, i.e., oral intercourse, anal intercourse, vaginal intercourse, penetration with finger and/or objects etc.).
